# Adulthood Exposure to Lipopolysaccharide Exacerbates the Neurotoxic and Inflammatory Effects of Rotenone in the Substantia Nigra

**DOI:** 10.3389/fnmol.2017.00131

**Published:** 2017-05-08

**Authors:** Chun Huang, Li Zhu, Huan Li, Fu-Guo Shi, Guo-Qing Wang, Yi-Zheng Wei, Jie Liu, Feng Zhang

**Affiliations:** Key Laboratory of Basic Pharmacology of Ministry of Education and Joint International Research Laboratory of Ethnomedicine of Ministry of Education, Zunyi Medical UniversityZunyi, China

**Keywords:** Parkinson’s disease, microglia, lipopolysaccharide, rotenone, neurotoxicity

## Abstract

Parkinson’s disease (PD) is the second most neurodegenerative disorder with a regional decrease of dopamine (DA) neurons in the substantia nigra (SN). Despite intense exploration, the etiology of PD progressive process remains unclear. This study was to investigate the synergistic effects of systemic inflammation of lipopolysaccharide (LPS) and neurotoxicity of rotenone (ROT) on exacerbating DA neuron lesion. Male SD adulthood rats received a single intraperitoneal injection of LPS. Seven months later, rats were subcutaneously given ROT five times a week for consecutive 4 weeks. Rat behavior changes were assessed via rotarod and open-field tests. Brain SN was immunostained to evaluate DA neuronal loss and microglia activation. Striatum DA and its metabolites levels were determined by high performance liquid chromatography (HPLC) coupled with electrochemistry. The protein levels of α-synuclein (α-Syn), inflammatory factors and mitogen-activated protein kinase (MAPK) pathway activation were detected by western blotting analysis. Results indicated that no significant difference between the control and LPS alone groups was shown. Compared with ROT alone group, LPS combined with ROT significantly reduced motor activity and induced SN DA neurons loss accompanied by the decreased contents of striatum DA and its metabolites. Furthermore, LPS together with ROT enhanced microglia activation and the increased expressions of α-Syn and inflammatory factors and also MAPK signaling pathway activation. However, LPS alone had no significant effects on the above parameters. These findings suggest that adulthood exposure to LPS exacerbates the neurotoxic and inflammatory effects of ROT in the SN.

## Introduction

Parkinson’s disease (PD), a progressive neurodegenerative disorder, is characterized by a gradual loss of dopamine (DA) neurons in the substantia nigra (SN). The pathological hallmark of PD is the formation of Lewy Body with the main component of α-synuclein (α-Syn) in mesencephalic DA neurons. At present, several available therapeutics only temporarily relieve symptoms of this disorder, but no effective therapy is available to reverse its progression. Until now, the etiology of PD and especially the mechanisms underlying the gradual loss of DA neurons are poorly understood.

Recently, increasing evidence supported the role of neuroinflammation in the pathogenesis of PD. Microglia activation and the subsequent release of microglial pro-inflammatory factors are closely associated with the progressive degradation of DA neurons in the midbrain of PD patients (De Virgilio et al., 2016). Inhibition of pro-inflammatory factors production could reduce DA neuronal damage (Fu et al., 2015). Thus, the activation of microglia and the production of abundant inflammatory and cytotoxic factors, including cytokines, free radicals, might come into a driving force to keep successive vicious cycle of DA neuron injury (Gao et al., 2011b). It has been well demonstrated that the nigral infusion or intraperitoneal injection of the common inflammogen, lipopolysaccharide (LPS), resulted in time-dependent DA neurodegeneration in rodent SN (Ling et al., 2006; Gordon et al., 2016). However, the mechanisms how long-term neuroinflammation affected progressive DA neurodegeneration requires further investigation (Gao et al., 2011a).

In addition, a close relationship of environmental exposure with PD occurrence (Liu et al., 2003) has been manifested. Recent case-control and epidemiological evidence confirms that PD is positively correlated with lifetime use of the pesticide, rotenone (ROT; Tanner et al., 2011). In a population with well-characterized pesticide exposure, ROT reproduced parkinsonism features during chronically administrated to rodents (Cannon et al., 2009). Thus, ROT becomes an increased risk factor for PD (Zhou et al., 2012). Mechanistically, ROT damaged DA neuronal mitochondrial complex I (Jenner, 2001) and further disrupted the microtubule-based transport of neurotransmitter vesicles (Ren and Feng, 2007). Intensive studies demonstrate that ROT induced DA neurotoxicity and also microglia activation, suggesting neuroinflammation participated in ROT-induced DA neuron lesion (Gao et al., 2002).

Current consensus implies that PD processes from complex inflammation–environment interactions. Compared with a previous study presented ROT and LPS worked in synergy to induce a selective DA neurodegeneration, these findings were based on the cell culture of investigation and the *in vivo* observations warrant further elucidation (Gao et al., 2003). Furthermore, several interesting studies revealed that the combined effects of prenatal LPS and postnatal ROT exposure produced a synergistic DA neurotoxicity in female rats (Ling et al., 2004) and neonatal intracerebral injection of LPS enhanced ROT-induced SN DA neurotoxicity in rats (Fan et al., 2011). Furtherly, neonatally intraperitoneal injection of LPS strengthened susceptibility of nigral DA neurons to ROT neurotoxicity (Cai et al., 2013). However, the interaction between adulthood exposure to LPS and ROT on the pathogenesis of PD in rodent adulthood remains unknown.

In the present study, adulthood exposure to LPS combined with ROT-induced animal model was performed to investigate mechanisms underlying PD progression. Rats received a single intraperitoneal injection of LPS followed by ROT administration. Specifically, this study explored how adulthood exposure to LPS and environmental toxicant interacted to influence DA progressive neurodegeneration.

## Materials and Methods

### Reagents

LPS (0111:B4) and ROT were purchased from Sigma Chemical Co. (St. Louis, MO, USA). The standard products of DA and its metabolites, 3,4-dihydroxyphenylacetic acid (DOPAC) and homovanilic acid (HVA) were purchased from the National Institute for Food and Drug Control (China). Lysis Buffer and the enhanced chemiluminescence (ECL) reagent were purchased from Beyotime Institute of Biotechnology (Shanghai, China). Anti-α-Syn (Catalog No. 10842-1-AP, rabbit polyclonal), inducible nitric oxide synthase (iNOS; Catalog No.18985-1-AP, rabbit polyclonal), cyclooxygenase-2 (COX-2; Catalog No.12375-1-AP, rabbit polyclonal), interleukin-1β (IL-1β; Catalog No.16806-1- AP, rabbit polyclonal) and tumor necrosis factor-α (TNF-α; Catalog No.17590-1-AP, rabbit polyclonal) antibodies were purchased from Proteintech Group (Chicago, IL, USA). Anti-tyrosine hydroxylase (TH; Catalog No. ab415286806, rabbit polyclonal), CD11b/c (OX-42; Catalog No. ab1211, mouse monoclonal) and ionized calcium-binding adapter molecule-1 (Iba-1; Catalog No. ab15690, mouse monoclonal) antibodies were bought from Abcam (Cambridge, MA, USA). Anti-extracellular regulated protein kinases 1/2 (ERK1/2, Catalog No. mAb 4695, rabbit monoclonal), phosphorylated ERK1/2 (p-ERK1/2, Catalog No. mAb 4370, rabbit monoclonal), c-Jun N-terminal kinase (JNK, Catalog No. mAb 9252, rabbit monoclonal), phosphorylated-JNK (p-JNK, Catalog No. mAb 4668, rabbit monoclonal), phosphorylated-p38 (p-p38, Catalog No. mAb 4511, rabbit monoclonal), p38 (Catalog No. mAb 8690, rabbit monoclonal) and β-actin (Catalog No. mAb 4970, rabbit monoclonal) were purchased from Cell Signaling Technology (Beverly, MA, USA).

### Animals and Treatment

A total of 72 6-month-old Sprague Dawley male rats were obtained from the Experimental Animal Center of the Third Military Medical University (Chongqing, China; Specific-pathogen-free Grade II; Certificate No. SCXK 2012–0011). All animal experiments were performed in accordance with Chinese Guidelines of Animal Care and Welfare and the present study was approved by the Animal Care and Use Committee of Zunyi Medical University (Zunyi, China). This study was randomly divided into the control, LPS, ROT and LPS combined with ROT groups. Rats were given a single intraperitoneal injection of normal saline (NS) or LPS (5 mg/kg). Seven months later, rats received subcutaneous injection of ROT (0.5 mg/kg) or NS (an equivalent volume of ROT) five times a week for consecutive 4 weeks (Ojha et al., 2015). After the last treatment of ROT, rats were allowed to recover for an additional 2 weeks development followed by the behavior tests performed. At 15th day after the last ROT treatment, all animals were sacrificed under anesthesia for the biochemical analysis.

### Rotarod Test

Rotarod test was carried out for the study of muscular coordination. It consisted of cylindrical arrangement of thin steel rods, 75 mm in diameter, which was divided into two parts by compartmentalization to permit the testing of two rats at a time. In the train, the speed was set at 10 cycles per min and cut-off time was 180 s. Prior to the start of the test, rats in all groups were trained on rotarod until they stayed on the rod at least for the cut-off time (Khuwaja et al., 2011). Animals were allowed to remain stationary for a while at 0 rpm. The rotational speed was steadily increased to 10 rpm in 20 s interval till the rats fell off the rungs. Animals were tested for two trials per day and the mean duration time stayed on the rod was recorded (Hamm, 2001).

### Open-Field Test

The open-field apparatus (OFT) contained a round, 96 cm diameter arena surrounded by a 50 cm high fence and is divided into four parts which is painted black. These parameters were widely taken as indicative of high-stress state, thus, for the evaluation of levels of emotionality of the animals (Bergamini et al., 2017). For open-field observations, each rat was individually placed on the center of the arena, and its behavioral parameters were recorded for 10 min. The apparatus was washed with a 5% ethanol solution before each behavioral test to preclude the possible cueing effects of odors left by previous subjects. Control and experimental rats were intermixed to minimize the possible influences of circadian rhythmicity on rat behavior. The distance of center and periphery area were listed by the computer. After the observation period, the total travel distance in the arena of the OFT was counted (Santiago et al., 2014).

### High Performance Liquid Chromatography (HPLC) Coupled with Electrochemical Detection

Rat striatal levels of DA and its metabolites, DOPAC and HVA, were determined by high performance liquid chromatography (HPLC) coupled with electrochemical detection. Striatal tissues were sonicated in perchloric acid containing the internal standard 3,4-dihydroxybenzylamine (10 mg wet tissue/ml). The homogenate was centrifuged and an aliquot of the supernatant was injected into the HPLC equipped with a C_18_ column (Dionex, Germering, Germany). The mobile phase was comprised of acetonitrile, tetrahydrofuran and monochloroacetic acid (pH 3.0) including Ethylenediaminetetraacetic acid (EDTA, 50 mg/L) and sodium octyl sulfate (200 mg/L). The levels of DA, DOPAC and HVA were detected by comparison of peak height ratio of tissue sample with standards (Zhang et al., 2006).

### Immunohistochemistry Staining and Counting

Rat brains were cut into 35 μm transverse free-floating sections on a horizontal sliding microtome. A total of 36 consecutive brain slices throughout the entire SN was collected, and every 6th section was selected for the immunohistochemical staining. DA neurons and microglia were recognized through anti-TH (a marker of DA neuron) and OX-42 (a marker of activated microglia) antibodies, respectively. Briefly, formaldehyde (4%) fixed the brain slices were treated with 0.3% triton and 3% hydrogen peroxide followed by sequential incubation with blocking solution. Next, the slices were incubated overnight at 4°C with anti-TH (1:300) and OX-42 (1:300) antibodies. Subsequently, brain slices were incubated with biotinylated secondary antibody for 1 h and then the color was developed with 3,3′-diaminobenzidine. For the morphological analysis, the digital images of TH-positive neurons and OX-42-positive microglia in the SN were obtained via an Olympus microscope (Olympus, Tokyo, Japan) using an attached Polaroid digital microscope camera (Polaroid, Cambridge, MA, USA). The quantification of TH-positive neurons was performed through visually counting the number randomly by two testers, and the average was the results. The mean value for the number of SN TH-positive neurons was then calculated through averaging the counts of six sections for each animal.

### Western Blotting Analysis

The tissues of rat midbrain were collected and protein expressions were detected by western blotting assay. Briefly, the tissues were homogenized and lysed in radio-immunoprecipitation assay (RIPA) lysis buffer supplemented with proteinase inhibitors phenyl methyl sulfonyl fluoride (PMSF). RIPA cell lysis buffer consisting of 50 mM Tris-HCl (pH 7.4), 150 mM NaCl, 1 mM EDTA, 5 μg/ml Aprotinin, 1 mM PMSF, 5 μg/ml Leupeptin, 1% Triton X-100, 1% Sodium deoxycholate, 1 mM Na3VO4. The lysates were treated on ice for 30 min and centrifuged at 14,000 *g* for 15 min. The supernatant was acquired and the whole protein levels were quantified by BCA assay kit. Equal amounts of total protein (30–50 μg per lane) were separated on 10% Bis-Tris Nu-PAGE gel by electrophoresis and then transferred onto polyvinylidene difluoride (PVDF) membranes. Membranes were blocked with 5% nonfat milk and incubated overnight at 4°C with the following primary antibodies: anti-β-actin (1:500), TH (1:1000), Iba-1 (1:1000), α-Syn (1:500), iNOS (1:500), COX-2 (1:500), IL-1β (1:500), TNF-α (1:500), p-ERK1/2 (1:1000), ERK1/2 (1:1000), p-JNK (1:1000), JNK (1:1000), p-p38 (1:1000), p38 (1:1000) and horseradish peroxidase-conjugated secondary antibodies at 1:1000 dilution. The blots were visualized using the ECL reagent.

### Statistical Analysis

Data were expressed as mean ± standard error of the mean (SEM) from six rats. Statistical significance was assessed by one-way ANOVA using GraphPad Prism software (GraphPad Software Inc., San Diego, CA, USA). When ANOVA indicated the significant differences, pairwise comparisons between means were analyzed by Bonferroni’s *post hoc t* test with correction. A value of *p* < 0.05 was considered statistically significant.

## Results

### LPS and ROT Induced Behavioral Changes

To verify behavioral dysfunction in LPS and ROT-induced DA neuron lesion, we examined the motor-activity 14 days after the last injection of ROT. By the end of the experiment, rat body weight in each group was basically unaltered, with body weight of Control (540 ± 20 g), LPS alone (545 ± 20 g), ROT alone (520 ± 20 g) and LPS + ROT (510 ± 20 g) groups, indicating the animals were generally healthy.

**Figure 1:**
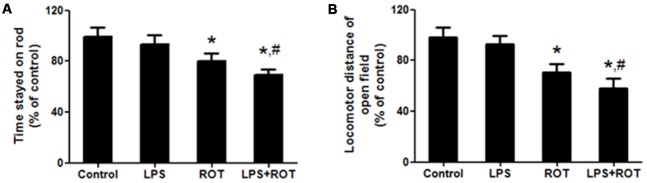
**Lipopolysaccharide (LPS) and rotenone (ROT) induced behavioral changes.** Rats were given a single intraperitoneal injection of LPS (5 mg/kg). Seven months later, rats received subcutaneous injection of ROT (0.5 mg/kg) five times a week for consecutive 4 weeks. After the last treatment of ROT, rats were allowed to recover for an additional 2 weeks development followed by the rotarod and open-field tests performed. The time stayed on rod **(A)** and the total distance traveled in open-field test **(B)** were tested. Data were expressed as a percentage of the control group and were the mean ± SEM from six rats. *p < 0.05 compared with control group, ^#^*p* < 0.05 compared with ROT alone group.

Rotarod test is a one of classic methods to measure the muscular coordination in PD rat model. As shown in Figure 1A, ROT alone decreased the time stayed on the rod compared with the control group, while LPS alone had no significant influence in the time stayed on the rod. However, LPS together with ROT significantly reduced the time stayed on the rod compared with ROT alone group.

The open-field test is another classic behavioral test to comprehensively evaluate spontaneous locomotion in PD model. As shown in Figure 1B, rats in the control group performed well. No significant difference of locomotor distance between the control and LPS alone groups was discerned. ROT alone and ROT combined with LPS decreased the locomotor distance, whereas ROT + LPS had more toxic effects on motor changes than ROT alone.

### LPS Potentiated ROT-Induced DA Neuronal Loss in the SN Region

**Figure 2:**
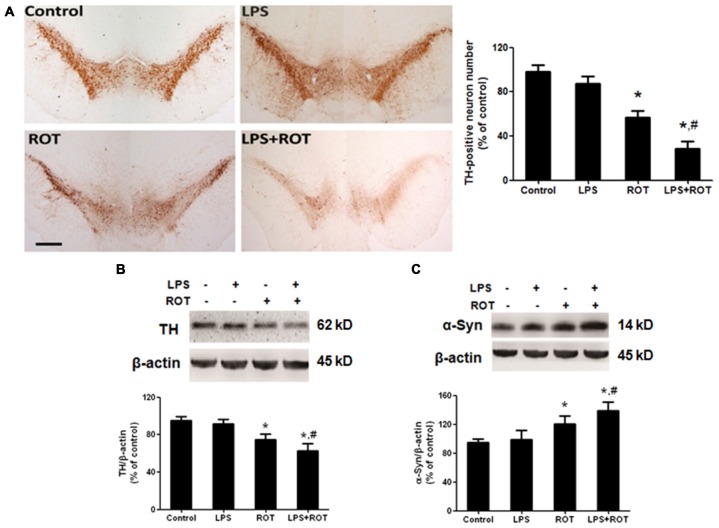
**LPS potentiated ROT-induced dopamine (DA) neuronal loss in the substantia nigra (SN) region.** Rats received a single intraperitoneal injection of LPS (5 mg/kg) for 7 months followed by the subcutaneous injection of ROT (0.5 mg/kg) five times a week for consecutive 4 weeks. Two weeks after the last ROT treatment, rats were sacrificed and DA neurons lesion was analyzed via the DA neuronal counting by immunostaining. The representative brain sections were immunostained with an anti-tyrosine hydroxylase (TH) antibody and the number of TH-positive neurons in the SN was counted **(A)** Scale bar = 200 μm. Rat mesencephalons were obtained and SN TH **(B)** and α-Syn **(C)** protein expressions were detected by western blotting assay. The ratio of densitometry values of TH and α-Syn with β-actin was analyzed and normalized to each respective control group. Data were expressed as a percentage of the control group and were the mean ± SEM from six rats. *p < 0.05 compared with the control group, ^#^*p* < 0.05 compared with ROT alone group.

After the behavior tests were finished, rats were sacrificed and brains were sectioned and performed for DA neurons quantification by immunostaining analysis with an anti-TH antibody. As shown in Figure 2A, ROT alone produced a approximately 40% loss of nigral TH-positive neurons compared with the control group, while no significant loss was indicated in LPS alone group. Moreover, a loss of TH-neurons reached 60% in LPS combined with ROT group. Similarly, the results of TH protein expression from western blotting assay were consistent with the immunocytochemistry results (Figure 2B).

To further observe the link of DA neuronal loss with aggregation of α-Syn in the SN, the effects of LPS and ROT on α-Syn protein expression were detected. As shown in Figure 2C, no significant difference of α-Syn protein expression between the control and LPS alone groups was presented. However, ROT alone increased α-Syn protein expression compared with the control group. Furtherly, the α-Syn protein expression in LPS together with ROT group was greater than that in ROT alone group.

### Alteration in Rat Striatum DA and its Metabolites Treated with ROT and LPS

**Figure 3:**
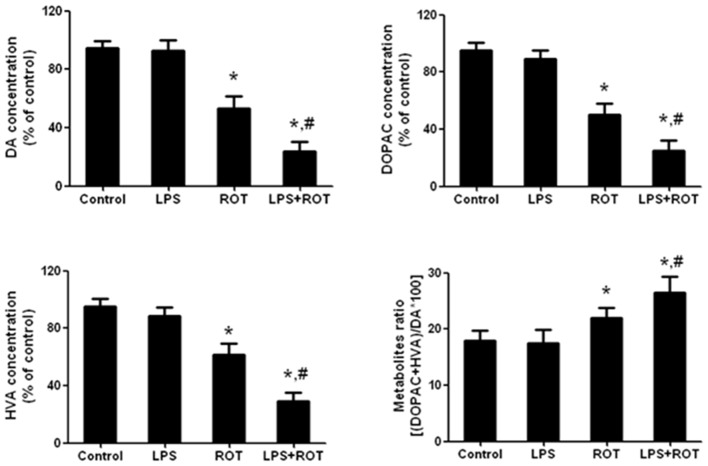
**Alteration in rat striatal DA and its metabolites treated with ROT and LPS.** A single LPS (5 mg/kg) was intraperitoneally injected into rats. Seven months later, ROT (0.5 mg/kg) was subcutaneously injected into rats five times a week for consecutive 4 weeks. Two weeks after the last treatment of ROT, the striatal tissue levels of DA, 3,4-dihydroxyphenylacetic acid (DOPAC) and homovanilic acid (HVA) were measured by high performance liquid chromatography (HPLC) coupled with electrochemical detection. The DA metabolite ratio ((HVA + DOPAC)/DA*100) was also calculated. Data were expressed as a percentage of the control group and were the mean ± SEM from six rats. *p < 0.05 compared with control group, ^#^*p* < 0.05 compared with ROT alone group.

Rat striatum was collected to perform DA, DOPAC and HVA detection. As shown in Figure 3, compared with the control group, ROT alone decreased rat striatal DA, DOPAC and HVA levels, whereas LPS alone had no significant effects on DA and its metabolites contents. Compared with ROT alone group, LPS combined with ROT significantly reduced DA, DOPAC and HVA levels in rat striatum with approximately 70%–80% decrease of control group. Moreover, a decreased turnover of DA metabolite ratio ((HVA + DOPAC)/DA*100) in ROT alone and LPS + ROT groups was discerned compared with control group.

### LPS Strengthened ROT-Induced Microglia Activation in SN

**Figure 4:**
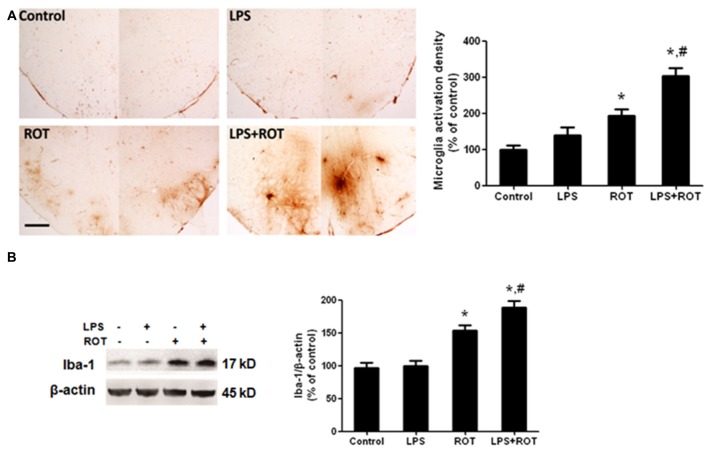
**LPS strengthened ROT-induced microglia activation in the SN.** Rats were administrated with a single intraperitoneal LPS (5 mg/kg) injection. Seven months later, rats received subcutaneous ROT (0.5 mg/kg) injection five times a week for 4 weeks. Two weeks later, rat SN microglia activation was analyzed through immunostaining and western blotting analysis. Rat brain sections were immunostained with anti-OX-42 antibody **(A)**. The densitometry analysis of SN OX-42-positive microglia was performed via ImageJ software. Scale bar = 200 μm. Rat mesencephalons were obtained and the Iba-1 protein level was determined by western blotting analysis **(B)**. The ratio of densitometry values of Iba-1 with β-actin was analyzed and normalized to each respective control group. Data were expressed as a percentage of the control group and were the mean ± SEM from six rats. *p < 0.05 compared with the control group, ^#^*p* < 0.05 compared with ROT alone group.

Rat brains were sectioned and microglia activation was evaluated via immunostaining with an anti-OX-42 antibody. As illustrated in Figure 4A, the resting microglia exhibited a characeristic ramified morphology in the control group. In ROT alone group, microglia were activated and characterized by larger size, thicker processes and more round status. Also, microglia activation and morphologic changes were significantly enhanced in LPS combined with ROT group. Moreover, the protein level of Iba-1, another marker of microglia activation, was detected by western blotting analysis. As revealed in Figure 4B, similar results were observed that LPS combined with ROT enhanced microglia activation compared with ROT alone group.

### Elevation of Inflammatory Factor Protein Expression and MAPK Pathway Activation in SN after LPS and ROT Treatment

**Figure 5:**
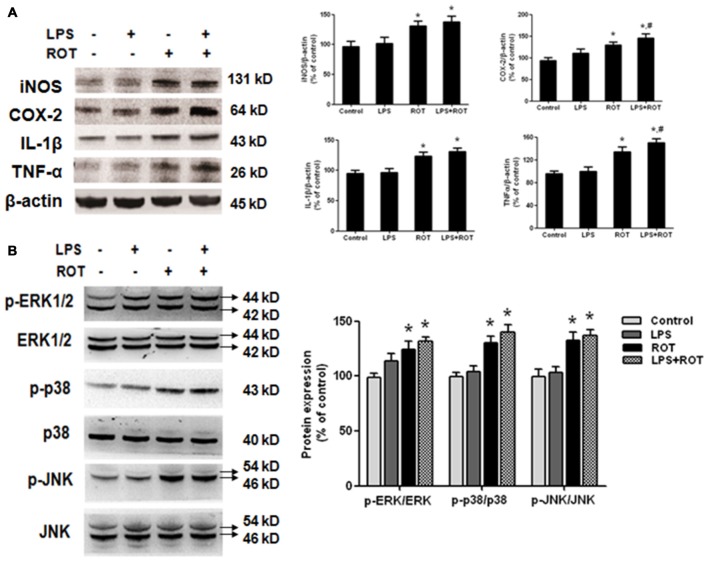
**Elevation of inflammatory factors protein expressions and mitogen-activated protein kinase (MAPK) pathway activation in SN after LPS and ROT treatment.** Rats got a single intraperitoneal injection of LPS (5 mg/kg). Seven months later, the subcutaneous injection of ROT (0.5 mg/kg) was applied in rats five times a week for 4 weeks. Two weeks later, rat mesencephalons were obtained and the protein levels of inflammatory factors **(A)**, such as IL-1β, COX-2, inducible nitric oxide synthase (iNOS) and TNF-α, and MAPK pathway activation **(B)** in SN were measured by western blotting assay. The ratio of densitometry values of IL-1β, COX-2, iNOS and TNF-α with β-actin, and p-ERK1/2, p-p38 and p-JNK with total ERK1/2, p38 and JNK, was analyzed and normalized to each respective control group. Data were expressed as a percentage of the control group and were the mean ± SEM from six rats. *p < 0.05 compared with the control group, ^#^*p* < 0.05 compared with ROT alone group.

The protein levels of inflammatory factors in SN were determined by western blotting assay. As shown in Figure 5A, no significant difference of inflammatory factors protein expressions in SN between the control and LPS alone groups was seen. On the contrary, ROT alone increased IL-1β, COX-2, iNOS and TNF-α protein expressions compared with the control group. Furtherly, LPS combined with ROT significantly increased the above inflammaotry factors protein expressions, particularly with the higher protein expressions of COX-2 and TNF-α than those in ROT alone group. Furtherly, compared with control group, ROT and LPS + ROT significantly enhanced ERK1/2, JNK and p38 protein phosphorylation, whereas LPS alone had no significant effects on mitogen-activated protein kinase (MAPK) pathway activation (Figure 5B).

## Discussion

The present study demonstrates that adulthood LPS exposure bridged ROT and progressive DA neurodegeneration in mediating chronic PD progression. LPS combined with ROT significantly reduced motor activity and induced SN DA neuronal loss accompanied by the decreased levels of striatum DA and its metabolites. In addition, LPS together with ROT enhanced microglia activation and increased expressions of α-Syn and inflammatory factors and also induced MAPK signaling pathway activation. These findings suggest adulthood exposure to LPS exacerbated the neurotoxic and inflammatory effects of ROT in the pathogenesis of PD.

PD is an aging-related neurodegenerative disease and its etiology is still unknown. A number of *in vivo* animal models attempt to capture as many of the hallmark of PD as possible. Several lines of evidence that contact with environmental toxicants, such as ROT, could be closely associated with an increased risk of PD, especially through gene–environment interactions (Brown et al., 2006; Wang et al., 2011). Studies from rodents experiments have begun to examine the ability of ROT and paraquat to mimic various aspects of PD, although the variability in phenotypes becomes a significant hurdle to overcome. ROT administration in rats has been confirmed to result in the nigral DA neuron lesions and α-Syn accumulation (Cannon et al., 2009), despite the lack of specificity of the toxin and the variability of its effects in earlier studies have limited its use for capturing all of the hallmarks of PD and further evaluating the potential neuroprotective strategies (Fleming et al., 2004; Zhu et al., 2004). Thus, a single given toxic insult model should never be applied indiscriminately to explore the underlying etiology of PD, but should instead be carefully selected on the basis of being the most suitable model (Frank-Cannon et al., 2008; Jackson-Lewis et al., 2012). In the present study, we found LPS strengthened ROT–elicited DA neuronal loss, demonstrating that the interactions between systemic inflammation and environmental toxicants on DA neurodegeneration warrant further investigation.

Growing evidence indicates that direct infection or hyper-sensitivity to foreign proteins might cause an intrinsic inflammation (de Groot and Burgas, 2015). In addition, brain is also vulnerable to damage in response to inflammation as infiltration of immune cells and mediators could generate profound structural and functional changes (Sankowski et al., 2015). The blood–brain barrier (BBB) separates the central nervous system (CNS) from the circulating blood, creating a privileged environment. Although this is indispensable for maintaining brain homeostasis, it didn’t mean that brain is depleted of immune cells (Amor et al., 2010). Furthermore, the CNS is not completely isolated from blood circulation since peripheral pro-inflammatory cytokines might by-pass the BBB in the circumventricular organs and mobilize and activate the resident macrophages (Perry, 2004).

Microglia, the resident macrophages of CNS, serve critical roles in the immune surveillance under resting conditions. Upon activated by brain damage or inflammogen stimuli, microglia release various pro-inflammatory and cytotoxic factors, such as cytokines, reactive oxygen species (ROS) and reactive nitrogen species (RNS). The accumulation of these factors is considered to contribute to the progressive loss of DA neurons (Block et al., 2007). However, the continuing dying/dead DA neurons also release kinds of neurotoxic soluble factors such as α-Syn and damage-associated molecular patterns (DAMPs), which in turn again elicited microglia activation (termed as reactive microgliosis). The reactivated microglia still produced pro-inflammatory factors and resulted in the continuous DA neuronal lesion (Chen et al., 2016). Therefore, a “self-propelling” vicious cycle was created to induce the chronic progressive DA neurodegeneration. In this study, ROT alone caused DA neuronal loss and indirectly induced microglia activation and subsequent release of pro-inflammatory factors, while LPS alone had no significant toxicity to DA neuronal survival. However, at the presence of LPS pretreatment, LPS seemed to enhance microglia activation along with ROT-produced DA neurotoxicity and consequently launch this vicious cycle running.

To further confirm LPS-enhanced ROT-induced neuroinflammatory response, the effects of LPS and ROT on MAPK signaling pathway activation were observed. Since the key signaling pathways mediating inflammatory responses, MAPK signals are responsive to different environmental stimuli and have been elucidated in various types of inflammatory diseases. Three members of the MAPK family, ERK1/2, JNK and p38, are all activated upon neuroinflammation, and inhibition of this pathway reduces the production of pro-inflammatory cytokines and exerts neuroprotection against the insult (Bhatia et al., 2016; Jeong et al., 2016). In this study, we found that both ROT alone and ROT together with LPS induced more significant activation of MAPK pathway than LPS treatment.

A host of studies indicated that a single nigral pathway injection of LPS led to the acute and dramatic neuroinflammatory reactions and DA neuronal loss accompanied by animal behavior changes, the decreased levels of striatal DA, DOPAC and HVA, nigral microglial activation and subsequent production of neuroinflammatory factors, such as such TNF-α, IL-1β and NO (Zhang et al., 2006, 2010). However, that acute model did not replicate a chronic and progressive course of PD. Either, the acute and located nigral inflammation might not be highly associated with the potential etiology and pathogenesis of PD. Interestingly, this study found that LPS alone failed to induce neuroinflammation and the subsequent SN DA neuronal lesion. These findings were consistent with previous studies that LPS-injected wild-type mice had no significant effects on chronic neuroinflammation development, α-Syn accumulation, and detectable DA neuronal loss in SN (Gao et al., 2011a). The correct mechanisms underlying this phenomenon warranted further investigation.

In conclusion, the intraperitoneal injection of LPS followed by subcutaneous injection of minimally toxic dose of ROT (0.5 mg/kg) demonstrated the accelerated DA neurons lesion. Thus, the present study advances exploration to illuminate a mechanistic link among adulthood LPS exposure, environment toxicant treatment and progressive DA neurodegeneration.

## Conclusion

Adulthood exposure to LPS exacerbated the neurotoxic effects of ROT in the SN through microglial activation and inflammatory factors release leading to the progressive DA neurodegeneration.

## Author Contributions

FZ and JL conceived and designed the experiments. All the authors participated in the experiments performance and data analysis. FZ, JL and CH wrote, revised and checked the article. All the authors have reviewed the contents of the manuscript and approved the submitted manuscript.

## Conflict of Interest Statement

The authors declare that the research was conducted in the absence of any commercial or financial relationships that could be construed as a potential conflict of interest.
